# The Role of Myelin in Theiler's Virus Persistence in the Central Nervous System

**DOI:** 10.1371/journal.ppat.0030023

**Published:** 2007-02-16

**Authors:** Jean-Pierre Roussarie, Claude Ruffié, Michel Brahic

**Affiliations:** Unité des Virus Lents, Département de Virologie, Institut Pasteur and Centre National de la Recherche Scientifique, Paris, France; University of California San Francisco, United States of America

## Abstract

Theiler's virus, a picornavirus, persists for life in the central nervous system of mouse and causes a demyelinating disease that is a model for multiple sclerosis. The virus infects neurons first but persists in white matter glial cells, mainly oligodendrocytes and macrophages. The mechanism, by which the virus traffics from neurons to glial cells, and the respective roles of oligodendrocytes and macrophages in persistence are poorly understood. We took advantage of our previous finding that the *shiverer* mouse, a mutant with a deletion in the myelin basic protein gene *(Mbp),* is resistant to persistent infection to examine the role of myelin in persistence. Using immune chimeras, we show that resistance is not mediated by immune responses or by an efficient recruitment of inflammatory cells into the central nervous system. With both in vivo and in vitro experiments, we show that the mutation does not impair the permissiveness of neurons, oligodendrocytes, and macrophages to the virus. We demonstrate that viral antigens are present in cytoplasmic channels of myelin during persistent infection of wild-type mice. Using the optic nerve as a model, we show that the virus traffics from the axons of retinal ganglion cells to the cytoplasmic channels of myelin, and that this traffic is impaired by the *shiverer* mutation. These results uncover an unsuspected axon to myelin traffic of Theiler's virus and the essential role played by the infection of myelin/oligodendrocyte in persistence.

## Author Summary

Theiler's virus persists in the central nervous system of mice and causes a chronic disease that resembles multiple sclerosis, a common demyelinating disease of humans. The virus infects neurons for one to two weeks, but later on it persists in the white matter, in oligodendrocytes and also in macrophages. Oligodendrocytes are the myelin-making cells of the central nervous system. Strikingly, in mice with a genetic defect of myelin, the virus infects neurons normally but is unable to persist. Understanding the reason for the lack of persistence in this mutant mouse should pinpoint an essential step in the complex process resulting in persistence. In this article, we show that resistance to persistent infection is not mediated by the immune system and is not due to inefficient viral replication in oligodendrocytes or macrophages. Instead, we show that virus transported in axons traffics into the myelin, and that this traffic is interrupted by the myelin mutation. This unsuspected axon to myelin traffic of Theiler's virus is necessary for viral persistence. Our results warrant looking for a similar phenomenon in other persistent infections of the nervous system, including in humans.

## Introduction

The mechanisms by which viruses escape immune detection and establish persistent infections are extremely diverse. Over the past years, much has been learned about various ways in which viruses manipulate the innate and the adaptive immune system to their advantage. However, establishing a persistent infection may require more than dealing directly with the immune system. In this article, we describe how the infection of myelin and oligodendrocytes by virions transported in the axons of infected neurons is a critical step in the establishment of a persistent infection of the central nervous system (CNS) by Theiler's murine encephalomyelitis virus (TMEV).

TMEV is a picornavirus of mouse, transmitted by the oral/fecal route, which causes a chronic neurological disease when it reaches the CNS [1]. Although CNS disease is rare in the wild, it can be obtained routinely in the laboratory after intracerebral inoculation. Once in the CNS, the virus causes an acute encephalomyelitis with infection of neurons, and to a lesser extent, of macrophages and astrocytes in gray matter [2]. This “early disease” lasts approximately 2 wk, after which the virus is either cleared by the immune response, or persists in the CNS if the animals are genetically susceptible. Persistence of the infection causes gait disorders and incontinence, also referred to as “late disease” [3]. Susceptibility to persistent infection is multigenic with a major effect of the *H2* locus [1]. The virus does not persist in neurons but in glial cells of the white matter of spinal cord, mainly macrophage/microglial cells and oligodendrocytes, the myelin-making cells, and to a lesser extent astrocytes [4,5]. This persistent infection of white matter is focal and is accompanied by chronic inflammation made of CD4+ and CD8+ T cells, of B cells, and of activated macrophages. Primary demyelination, with conservation of axons, is ubiquitous in these foci. However, some axonal damage, including in noninflamed white matter, has been documented [6]. Taken together, these clinical and pathological findings are very reminiscent of those of multiple sclerosis (MS) in human. As a result, the infection by TMEV of genetically susceptible mouse strains, such as the SJL/J and C3H strains, is a classical MS model [1].

CNS myelin, the main target in MS, is an extension of the cytoplasmic membrane of the oligodendrocyte that wraps itself many times around axons. Most of the cytoplasm is extruded from myelin; however, cytoplasmic channels remain that connect the myelin sheath to the oligodendrocyte cell body. These channels contact the axon at the level of nodes of Ranvier, forming the so-called paranodal loops, as well as along the internode (see Figure S3) [7]. Several structural proteins, in particular myelin basic protein (MBP) and proteolipid protein (PLP) are important for the development and maintenance of myelin. MBP binds to the cytoplasmic surface of the myelin leaflets and is thought to play a role in myelin compaction. Due to the presence of more than one promoter and of alternate splicing, the *Mbp* gene codes for several isoforms of the protein [8]. Some of them are expressed in the immune system, in particular in T cells and macrophages. The BG21 isoform seems to downregulate T-cell activation [9]. PLP is an abundant integral membrane protein of myelin that may play a role in membrane apposition [10].

The *shiverer* mutation is a large deletion of the *Mbp* gene [11] that causes an extremely severe reduction of the amount of myelin in the mutant [12–14]. Several years ago we observed that C3H mice homozygous for the *shiverer* mutation were completely resistant to persistent infection by TMEV, whereas wild-type C3H mice were susceptible. In *shiverer* mice inoculated intracranially, TMEV infects neurons in the gray matter for about 10 d and causes an “early disease” very similar to that of wild-type mice. However, the virus then disappears from the CNS instead of persisting. Another myelin mutant, the *rumpshaker* mouse, which bears a point mutation in the *Plp* gene, is also resistant to persistent infection. For both mutants, resistance cannot be overcome by increasing viral dose [15]. The extreme resistance of *shiverer* and *rumpshaker* mice indicated that the *Mbp* and *Plp* mutations interacted with essential steps in the pathway leading to viral persistence. The experiments described in this article focused on the *shiverer* mutant and were designed to identify this step, thereby gaining new insights into the complex mechanisms that lead to the persistence of a picornavirus in CNS white matter and to a disease very similar to MS. Our results show that the infection of myelin cytoplasmic channels and of oligodendrocytes by virus coming from axons of infected neurons is critical for the establishment of persistent infection. We present several hypotheses concerning the role of myelin in persistence.

## Results

### The Resistance of *shiverer* Mice Is Not Mediated by Radiosensitive Bone Marrow–Derived Cells

**Figure 1:**
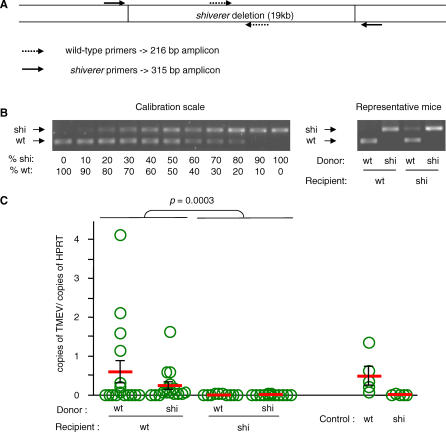
Susceptibility to Persistent Infection of Immune Chimeras between Wild-Type and *shiverer* Mice Recipient mice were irradiated at 1,100 rad, reconstituted with 2.5 × 10^6^ bone marrow cells from donor mice. (A) The degree of chimerism was evaluated on peripheral blood DNA using PCR and two pairs of primers located as shown. (B) The assay was calibrated using a series of reciprocal mixtures of wild-type and *shiverer* DNA. The result of the assay for four representative chimeras is shown on the right. Chimerism was at least 85% for all the mice used in the experiment. (C) The immune chimeras were inoculated with 10^6^ PFU of TMEV. The spinal cords were dissected out and the viral loads were measured by quantitative RT-PCR using HPRT mRNA as a reference. The ordinate shows the ratio (amount of viral RNA)/(amount of HPRT mRNA). Mice with a wild-type background and either a wild-type or a *shiverer* immune system were prone to persistent infection, whereas mice with a *shiverer* background and either a wild-type or a *shiverer* immune system were never persistently infected (Mann-Whitney test, *p* = 0.003). The results for age-matched wild-type and *shiverer* mice are shown as a control. There was no statistically significant difference between viral loads for control wild-type mice and wild-type recipients of wild-type bone marrow. Each circle corresponds to an individual mouse. wt, wild-type; shi, *shiverer.*

The golli isoforms of MBP (BG21 and J37) are expressed in the immune system, in particular in T-lymphocytes. BG21 seems to be a negative regulator of T-cell activation [9,16]. The function of J37 is less clear. Although BG21 is still expressed in *shiverer* mice, J37 is not. Therefore, the *shiverer* mutation may affect the adaptive immune responses against TMEV and allow the virus to persist in the CNS. To test for this possibility, we constructed immunological chimeras between wild-type (C3H+/+) and *shiverer* (C3H*shi/shi)* mice. Mice were lethally irradiated and their immune systems were reconstituted with autologous or heterologous bone marrow cells. The degree of chimerism of the PBLs was examined by PCR 7 wk after grafting. Two pairs of PCR primers were used. One, located within the *shiverer* deletion, amplified wild-type DNA only. The other pair spanned the large deletion and amplified mutant DNA only (Figure 1A). Figure 1B shows representative results obtained with four different mice. At least 85% of PBLs of all mice used were from the bone marrow donor. Age-matched, bone marrow–grafted, as well as control wild-type and *shiverer* mice, were inoculated intracerebrally with 10^6^ plaque-forming units (PFU) of TMEV. Viral loads in spinal cord were measured 45 d postinoculation (p.i.). The results for control mice showed the expected susceptibility and resistance of, respectively, wild-type and *shiverer* mice (Figure 1C, Table S1). Figure 1C also shows that susceptibility to persistent infection was not determined by the origin of bone marrow cells in chimeric mice. The *shiverer* mice that received wild-type bone marrow remained resistant, whereas the wild-type mice that received *shiverer* bone marrow remained susceptible. Therefore, the radiosensitive immune cells of *shiverer* mice are not responsible for resistance to persistent infection. This is in sharp contrast to the resistance of other resistant strains, such as the C57BL/6, which is due to viral clearance by the adaptive immune responses. Microglial cells, the resident CNS macrophages, secrete cytokines and chemokines and play an important role in the recruitment of inflammatory cells to the site of infections [17]. Because they turn over very slowly, if at all, they are not exchanged by bone marrow grafting [18]. Since they express some MBP isoforms, including J37 [19], they could be involved in the resistance of the *shiverer* mouse. To test for this possibility, wild-type and *shiverer* mice were injected intracranially with 10 μg of polyinosinic:polycytidilic acid [poly(I:C)], a double-stranded RNA mimic that activates microglial cells by binding to toll-like receptor 3 (TLR3) [20]. Inflammatory cells were extracted from the CNS 18 h later and analyzed by flow cytometry for the expression of CD11b, a marker of cells of monocytic origin, and CD45, an activation marker for these cells [21].The results showed that the percentage of activated cells of monocytic origin was the same for wild-type and *shiverer* mice (Figure S1A). Neurons and astrocytes can express TLR3 and can be a source of interferon during CNS viral infections [22–24]. However, since they do not express MBP, it is unlikely that the resistance of *shiverer* mice was due to an increase of interferon secretion by these cells.

Lastly, we compared the inflammatory cells present in the brain of wild-type and *shiverer* mice, 5 d p.i. with TMEV. Inflammatory cells were stained for CD3, CD4, and CD8 to study T-lymphocytes, and for CD19 and CD11c to study B cells and dendritic cells, respectively. The results of flow cytometry measurements are shown in Figure S1B. The frequency of each cell type was the same for wild-type and *shiverer* mice.

Therefore, the *shiverer* mutation does not affect the activation of microglial cells, the recruitment of inflammatory cells to the CNS following infection, and more generally, the adaptive, bone marrow–mediated immune response to the virus.

### The Early Disease Is Not Altered by the *shiverer* Mutation

**Figure 2:**
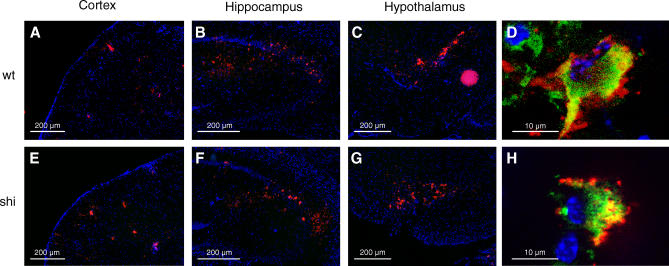
Viral Antigens in the Brain of Wild-Type and *shiverer* Mice Mice were inoculated intracranially with 10^6^ PFU of TMEV. The brain was dissected out 5 d p.i., snap-frozen, and 10-μm cryostat sections were cut. Sections were stained with an anti-NeuN antibody (green) and an anti-TMEV capsid serum (red). Nuclei were stained with DAPI. (A–D) Wild-type mice. (E–H) *shiverer* mice. The virus was predominantly found in cortex (A and E), hippocampus (B and F), hypothalamus (C and G), and thalamus (not shown). The distribution was the same in wild-type and in *shiverer* mice. (D and H) Examples of infected neurons in, respectively, wild-type and *shiverer* mice. Optical sections were obtained with the Zeiss ApoTome device. wt, wild-type; shi, *shiverer.*

Since the immune response was not responsible for the clearance of the virus by *shiverer* mice, we considered the possibility that the mutation impaired an important step of the viral life cycle in the CNS. A time course experiment showed that the viral load in brain and spinal cord was the same for wild-type and *shiverer* mice up to day 7 p.i. and that the virus disappeared from the CNS of the mutant between day 7 and day 11 p.i. (Figure S2 and Table S2). To look for differences in the pattern of CNS cell infection, coronal frozen sections of the temporal region of brain were reacted with fluorescent antibodies against the viral capsid, and serial sections were examined with a fluorescent microscope (Figure 2). The virus was present mainly in cortex (Figure 2A and 2E), hippocampus (Figure 2B and 2F), and hypothalamus (Figure 2C and 2G). The pattern was the same for wild-type and mutant mice. The nature of infected cells was examined using two-color immunofluorescence. Colocalization of fluorescent markers was assessed on optical sections obtained with the Zeiss Apotome fluorescent microscope. A systematic survey of infected cells showed that the majority of them, both in wild-type and in *shiverer* mice, expressed the neuron-specific NeuN marker. Figure 2D and 2H show examples of infected neurons in both cases.

**Figure 3:**
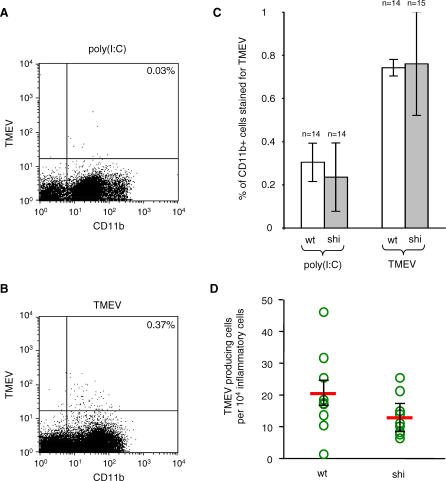
Infection of Brain Macrophages during Early Disease Wild-type and *shiverer* mice were inoculated intracranially with 4 × 10^6^ PFU of TMEV. The brain was dissected out 5 d p.i., mechanically and enzymatically dissociated, and inflammatory cells were purified by centrifugation on a Percoll gradient. Inflammatory cells were stained for CD11b and TMEV capsid antigens and analyzed by flow cytometry. Cells extracted from mice injected with poly(I:C) were used as negative controls for virus staining. (A) Representative analysis for a poly(I:C)-injected mouse. (B) Representative analysis for a TMEV-inoculated mouse. (C) Results obtained with 28 wild-type and 29 *shiverer* mice. The number of CD11b+ cells stained for capsid antigens was not statistically different between the two kinds of mice (Mann-Whitney test, *p* > 0.05). (D) Inflammatory cells were plated on a monolayer of BHK-21 cells and the number of viral plaques obtained was counted to measure the number of productively infected macrophages. No statistically significant difference was observed between wild-type and *shiverer* mice (Mann-Whitney test, *p* > 0.05). Each circle corresponds to an individual mouse. wt, wild-type; shi, *shiverer.*

Macrophages, derived from infiltrating monocytes or from activated microglia, are a major viral reservoir during persistent infection. Because they are also infected during early disease [2], it was possible to compare the permissiveness to TMEV of CNS macrophages in vivo in wild-type and mutant mice, 5 d after intracranial inoculation. This was fortunate, since permissiveness of primary macrophages and of macrophage cell lines is notoriously difficult to control and depends largely on culture conditions [25]. Inflammatory cells were extracted from the brain, labeled with anti-CD11b and anti-viral capsid antibodies, and analyzed by two-color flow cytometry. CD11b is expressed by infiltrating monocytes/macrophages and by resting as well as activated microglial cells [21]. Mice injected intracranially with 25 μg of poly(I:C) and sacrificed 18 h later served as controls. The poly(I:C)-induced inflammatory cells were used to set the threshold for detection of viral capsid antigens. Figure 3A and 3B shows an example of dot plot obtained in this experiment. The intensity of fluorescence for viral antigens was highly variable from cell to cell, probably reflecting various stages of the viral cycle. As can be seen in Figure 3C, the percentage of CD11b+ cells that were TMEV positive was not statistically different between wild-type and *shiverer* mice.

This experiment did not differentiate between infected macrophages, in which virus replicated, and macrophages ingesting infected cells and cellular debris. To distinguish between the two, we measured the production of infectious virus by CNS macrophages using an infectious center assay. Inflammatory cells were obtained as described above for flow cytometry. Serial 10-fold dilutions of cells were plated on monolayers of indicator BHK21 cells. The number of plaques obtained per macrophage for eight wild-type and eight *shiverer* mice is shown in Figure 3D. Although there is a trend for *shiverer* macrophages to produce fewer infectious centers, the difference was not statistically significant. Taken together, the results showed that the permissiveness of macrophages was the same in wild-type and in mutant mice.

### The *shiverer* Mutation Does Not Alter the Permissiveness of Oligodendrocytes to TMEV

**Figure 4:**
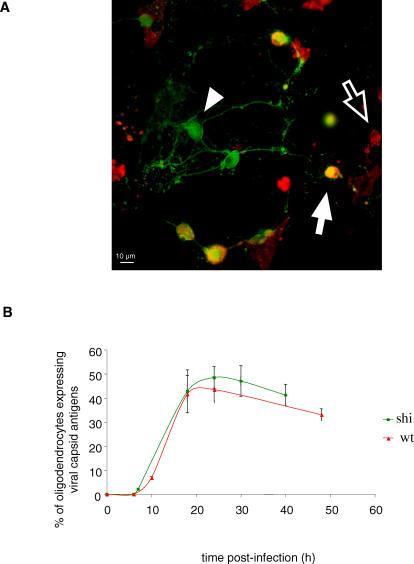
Infection of Primary Cultures of Oligodendrocytes from Wild-Type or *shiverer* Mice Cells were grown for 7 d with PDGF and 14 d without PDGF to induce oligodendrocyte differentiation. The cultures were infected with 500 PFU/cell of TMEV, fixed at different time points, and stained for CNPase, an oligodendrocyte marker (green) and viral capsid antigens (red). (A) Representative field of a wild-type culture 24 h p.i. White arrow, oligodendrocyte expressing viral capsid antigens; arrowhead, oligodendrocyte that did not express viral capsid; empty arrow, infected cell that was not an oligodendrocyte. (B) Percentage of oligodendrocytes that expressed viral capsid as a function of time p.i. There was no significant difference between wild-type and *shiverer* oligodendrocytes (Mann-Whitney test, *p* > 0.05, 18 and 24 h p.i.). wt, wild-type; shi, *shiverer.*

MBP, a major myelin protein, is expressed by oligodendrocytes. Since these cells are infected during late disease, we considered the possibility that the *shiverer* mutation could alter their permissiveness to the virus. Oligodendrocytes are not infected during early disease [2]; therefore, comparing the permissiveness of wild-type and *shiverer* oligodendrocytes could not be done in vivo, as was done for macrophages. Instead, we prepared primary cultures of oligodendrocytes from both types of mice and infected them in vitro at a multiplicity of infection of 500 PFU/cell. Because it is impossible to obtain oligodendrocyte cultures that are entirely devoid of astrocytes, and because astrocytes in culture are permissive to TMEV, it was not possible to compare viral titers. Instead we used two-color immunofluorescence to characterize the infection. Cells were fixed at various times p.i. and reacted with an anti-2′,3′-cyclic nucleotide 3′ phosphohydrolase (CNPase) antibody (an oligodendrocyte and myelin-specific marker) and an anti-viral capsid hyper immune serum. Infected oligodendrocytes were identified by colocalization of both markers (Figure 4A), and the percentage of CNPase-positive cells that expressed viral capsid antigens was measured (Figure 4B). The figure shows that the variation of this percentage with time was the same for both types of oligodendrocytes. The shape of the curve indicates that a single cycle of viral infection was achieved in approximately half the oligodendrocytes in the culture. Although the difference was not statistically significant, a slightly larger proportion of *shiverer* oligodendrocytes were infected, indicating that, if anything, *shiverer* oligodendrocytes might be slightly more permissive than wild-type oligodendrocytes.

### Myelin Contains Viral Antigens during Persistent Infection

**Figure 5:**
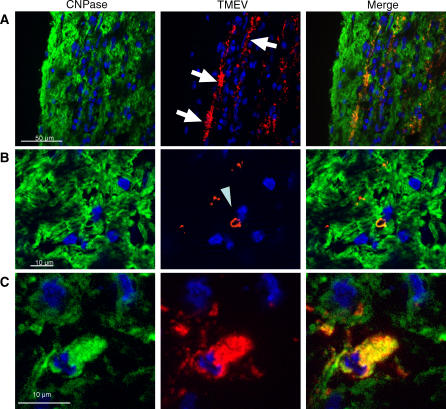
Viral Capsid Antigens in Myelin and Oligodendrocyte Cell Bodies during Persistent Infection Wild-type mice were inoculated intracranially with 10^6^ PFU of TMEV. Spinal cords were dissected out 45 d p.i., snap-frozen, and 10-μm cryostat sections (longitudinal or transverse) were cut. The sections were stained for the oligodendrocyte marker CNPase (green) and for viral capsid antigens (red). The nuclei were stained with DAPI (blue). Viral antigens were found to colocalize to a large extent with CNPase. (A) Longitudinal section. The linear pattern of capsid antigen (arrows) corresponds most probably to virus replication in myelin sheaths (see B and C). (B) Capsid antigens in myelin sheath as seen in transverse sections (arrowhead). (C) Capsid antigen in the CNPase-positive cell body of an oligodendrocyte.

Although most of the myelin sheath consists of juxtaposed plasma membranes, myelin also contains cytoplasmic channels, some of which, the paranodal loops and the inner loop, are in intimate contact with the axon (Figure S3). Interestingly, there is evidence that these channels may contain TMEV capsid antigens during persistent infection [5]. We re-assessed this notion using immunofluorescence and Apotome microscopy. Longitudinal and transverse frozen sections of the spinal cord of persistently infected C3H mice were reacted with an anti-CNPase antibody, an anti-viral capsid serum, and di-aminido phenylindol (DAPI), a nuclear stain. As shown in Figure 5A, capsid antigens often formed linear patterns with the same longitudinal orientation as axons. This pattern has been observed previously for both viral RNA and viral antigens (unpublished data). In double-labeled sections, viral antigens and CNPase colocalized in these patterns. Colocalization was more easily appreciated in transverse sections in which viral antigens and CNPase sometimes formed ring structures surrounding axons (Figure 5B). Such rings may correspond to paranodal loops or to the inner or the outer loop (Figure S3), which spiral around the axon. Capsid antigens were also observed in the CNPase-positive cell bodies of oligodendrocytes (Figure 5C). These results confirmed that myelin sheaths may contain viral capsid antigens during persistent infection. They indicate that the linear pattern of viral RNA and antigens observed during persistent infection of white matter is due the infection of myelin, more than to the presence of viral particles in axons.

### Virus Transported in the Axons Is the Source of Infection of Myelin and Oligodendrocytes

**Figure 6:**
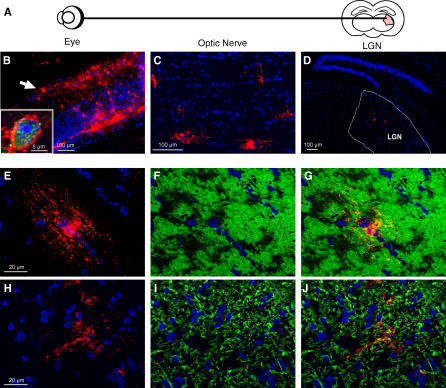
TMEV Infection in the Optic Tract Wild-type mice were inoculated intravitreously with 10^6^ PFU of TMEV. (A) Cartoon of the optical tract. (B) Cryostat sections of the retina stained for capsid antigens (red) and with DAPI (blue), 4 d p.i. The virus replicates in the retina, in particular in the ganglion cell layer (arrow). Insert: An infected retinal ganglion cell stained for NeuN (green) and viral capsid antigens (red). (C) TMEV capsid antigens (red) in a section of the ipsilateral optic nerve, 4 d p.i. The virus is present in glial cells along the nerve. (D) Coronal section of the brain of a wild-type mouse, 5 d p.i, stained for viral capsid antigens (red). The virus reached the LGN by axonal transport. (E–J) Optic nerve 4 d p.i. (E and H); Viral capsid antigens (red); (F) CNPase (green); (I) GFAP (green); (G) Merge of (E) and (F); and (J) Merge of (H) and (I). The virus infects oligodendrocytes and astrocytes.

Since the virus infects neurons during early disease and is transported axonally [26,27], myelin might be exposed to axonally transported virus, and the infection could spread secondarily to the cell body of the oligodendrocyte. Alternatively, oligodendrocyte cell bodies could be infected first, by virions diffusing from infected neurons, or more indirectly via the blood stream, and the infection could spread outwardly to myelin sheaths. To differentiate between these possibilities, we took advantage of the anatomy of the retina and of the optic nerve, both integral parts of the CNS. Ganglion cells of the retina, which are adjacent to the vitreous chamber, send their axons caudally through the optic nerve where they are myelinated by oligodendrocytes. The optic nerve does not contain neuron cell body; it contains only axons, oligodendrocytes, astrocytes, and microglial cells. We reasoned that if ganglion cells could be infected by virus injected in the vitreous chamber of the eye, and if the virus were transported in optic nerve axons, it should be possible to follow the spread of the virus from axons to glial cells in vivo in a fully compartmented system. Axonal transport could be assessed by looking for the infection of neurons in the lateral geniculate nucleus (LGN) to which ganglion cells project (see Figure 6A). By infecting one eye only, the contralateral optic nerve could serve as a control for axonal versus hematogenous source of virus. Ganglion cells from one side project to the LGN of the contra- or the ispsilateral side because of axon decussation at the chiasma. Therefore, only the pre-chiasmatic section of the optic nerve was examined. Wild-type mice were inoculated in the vitreous chamber of the right eye with 10^6^ PFU of TMEV, as described in Materials and Methods. Mice were sacrificed every day until day 5 p.i., and the eyes, the optic nerves, and the brain were prepared for immunohistological examination. Serial sections of the retina were stained for viral capsid antigens and nuclei were stained with DAPI. As shown in Figure 6B, infected cells were conspicuous in the retina. Cells in the intermediate layer were the first to be infected. Later, the virus spread to the layer of ganglion cells. The insert in Figure 6B shows that infected cells were neurons, as demonstrated by double staining with the NeuN antibody.

To assess axonal transport in the optic nerve, serial-frozen coronal sections of brain were prepared at the level of the thalamus and reacted with the anti-capsid serum and DAPI. The LGN was located and scanned systematically to record the presence of infected neurons. Viral antigens appeared in the LGN and nowhere else in thalamus, between days 3 and 5 p.i., demonstrating axonal transport. Figure 6D shows an example of infected cells in LGN, 5 d p.i.

Finally, serial-frozen sections of the pre-chiasmatic segment of the ipsi- and contralateral optic nerves of the same mice were stained for viral capsid antigens and for CNPase (oligodendrocytes) or glial fibrillary acidic protein (GFAP) (astrocytes). Viral antigens were first detected at day 4 p.i. Figure 6E–6G shows an example of a section of the ipsilateral optic nerve stained for the CNPase and viral markers. Viral antigens were observed in the cell bodies and cellular extensions of oligodendrocytes as well as in myelin. For each infected cell encountered, colocalization was assessed by scanning optical sections, 0.7 μm thick, obtained with the Apotome microscope. An example of a series of optical sections of an infected oligodendrocyte is shown in Video S5. The distribution of infected cells along the optic nerve appeared random (Figure 6C). There were no more infected cells toward the retina than toward chiasma making it very unlikely that the virus had diffused from the infected retina along the optic nerve. Figure 6H–6J and Video S6 show that some infected cells were astrocytes. In sharp contrast, no viral antigen was detected in the contralateral pre-chiasmatic optic nerve. Therefore, the source of infection of myelin and oligodendrocytes and astrocytes in optic nerve is virus-transported in axons.

### Myelin Is the Portal of Entry of the Virus into White Matter Oligodendrocytes

The experiments described so far suggest that the virus traffics from the axon to myelin cytoplasmic channels and from there to the oligodendrocyte cell body. Alternatively, oligodendrocyte cell bodies could be infected by virus diffusing from degenerating axons, and the infection could spread outwardly from the cell body to the myelin. We took advantage of the *shiverer* mouse mutant to distinguish between the two possibilities. Oligodendrocyte cell bodies are present in normal numbers in this mutant, but the amount of myelin is considerably reduced. We reasoned that if myelin is required for the infection of oligodendrocytes, the number of infected oligodendrocyte cell bodies should be reduced in *shiverer* mice.

**Figure 7:**
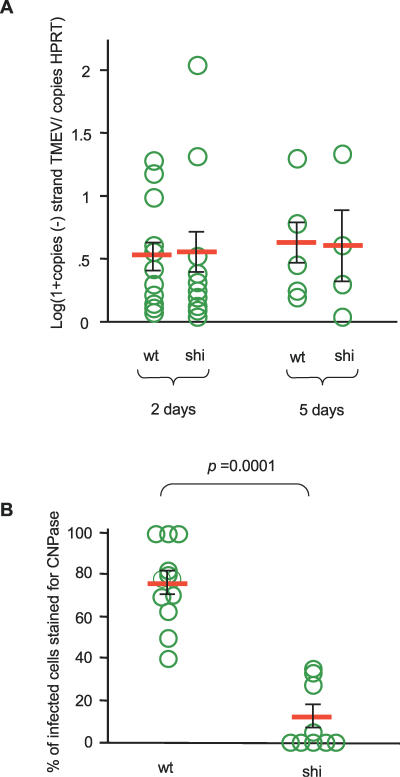
Infection of Retina and of Optic Nerve Oligodendrocytes in Wild-Type and *shiverer* Mice Wild-type and *shiverer* mice were injected intravitreously with 10^6^ PFU of TMEV. (A) Total RNA was extracted from the eye 2 d and 5 d p.i. The numbers of viral genomes of negative polarity (replicating RNA) and the number of HPRT mRNA were measured by RT-PCR. The ordinate shows the logarithm of the ratio (amount of minus-strand viral RNA)/(amount of HPRT mRNA). The replication of the virus inside the retina was the same in wild-type and *shiverer* mice. (B) Longitudinal sections of optic nerves obtained 4 d p.i. were scanned systematically under a fluorescence microscope equipped with the ApoTome device to assess marker colocalization. Oligodendrocytes were identified with an anti-CNPase antibody, astrocytes with an anti-GFAP antibody, and infected cells with an anti-capsid hyper-immune serum. The number of infected cells with an oligodendrocyte or an astrocyte phenotype was recorded. The ordinate shows the percentage of infected cells expressing CNPase. Each circle represents a different animal. The majority of infected cells in wild-type mice are oligodendrocytes. Conversely, most infected cells in *shiverer* mice are astrocytes. wt, wild-type, shi, *shiverer.*

In a first step, we compared the level of virus replication in retina, in wild-type and *shiverer* mice, 2- and 5-d post-intravitreous inoculation. Total RNA was extracted from the eye, and the level of minus-strand viral RNA was measured by real-time PCR. Minus-strand viral RNA is found only in infected cells. Therefore, this assay was not biased by viral particles from the inoculum. Figure 7A shows that virus replication was very similar for the two kinds of mice. In a second step, we compared the time of arrival of the infection in the LGN for wild-type and mutant mice using immunofluorescence as shown above and found no difference (unpublished data). Therefore, we concluded from the results of these controls that the amount of virus transported in the optic nerve must be very similar in wild-type and *shiverer* mice. We then systematically scanned frozen sections of optic nerves doubly stained for viral capsid antigens and CNPase, or for viral capsid antigens and GFAP, and the phenotype of each infected cell was recorded. A total of 12 wild-type and nine *shiverer* mice were examined. As shown in Figure 7B, 50%–100% of infected cells were oligodendrocytes in C3H wild-type mice. In contrast, no infected oligodendrocytes were found in six out of nine C3H *shiverer* mice. In the other three, only 30% of infected cells were oligodendrocytes. Therefore, the *shiverer* mutation considerably hampered the infection of oligodendrocytes by axonally transported virions. The axons in the optic nerve of the *shiverer* mouse are not entirely naked, but may have a single, or a few, turn(s) of myelin around them [28]. This may explain the small number of oligodendrocytes that were infected in *shiverer* optic nerves.

In summary, our results show that myelin is infected by axonally transported virus and that the infection spreads secondarily from the myelin to the oligodendrocyte cell body. This traffic is interrupted by the *shiverer* mutation. Since this is the only difference in the viral life cycle observed in this mouse mutant that is resistant to persistent infection, we infer that infecting myelin cytoplasm is essential for the persistence of Theiler's virus in the CNS.

## Discussion

Pathogenesis consists of an extremely large number of molecular interactions between the pathogen and its host. Genetic approaches, using host as well as pathogen mutants, are among the most powerful tools to identify essential steps in such a complex situation. Our previous, unexpected observation that *shiverer* and *rumpshaker* myelin-mutant mice were totally resistant to the persistent infection of the CNS by TMEV suggested to us that myelin may play a critical role in the establishment or the maintenance of the persistent infection. In the present work, we investigated the mechanism of resistance of the *shiverer* mutant with the hope of uncovering a hitherto undescribed step of pathogenesis, and we showed that the infection of myelin and oligodendrocytes from the axons of infected neurons is essential for viral persistence.

**Figure 8:**
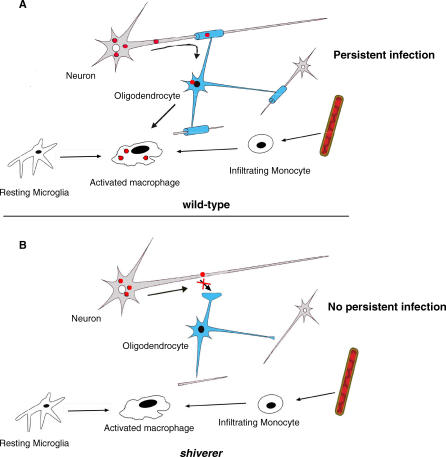
Cartoon of TMEV Pathogenesis The virus infects neurons in brain and spinal cord and is transported axonally during early disease. From the axon, it spreads to the surrounding cytoplasmic channels of myelin and from there to the cell body of oligodendrocytes. Macrophages, the main reservoir during persistent infection, are infected by virions released from infected oligodendrocytes, or by ingesting infected myelin. Macrophages can be activated infiltrating monocytes or activated microglial cells, or both.

Resistance to viral infections is often mediated by immune responses. Using immune chimeras and other tools, we found that this was not the case for TMEV persistence in the CNS. Therefore, we decided to follow the virus in the CNS, step by step, after intracranial inoculation. We observed that neither the early infection of neurons nor the permissiveness of oligodendrocytes and macrophages, the target cells during persistence, was affected by the *shiverer* mutation. We confirmed an observation made by electron microscopy decades ago, namely that the cytoplasmic channels of myelin are a site of viral expression during persistence [5]. We took advantage of the anatomy of the optic tract, from retina to LGN, to examine viral traffic between neurons, the main target during early disease, and glial cells. We showed that TMEV traffics from axons to myelin and oligodendrocyte cell bodies and that the *shiverer* mutation, which renders mice resistant to persistent infection, interrupts this traffic (Figure 8). Interestingly, Tsunoda and Fujinami proposed a similar “inside-out” model of viral spread from myelin to oligodendrocyte, based on entirely different considerations [6,29].

Why should the infection of myelin be important for the persistence of TMEV? TMEV replicates and spreads continuously through the CNS, although at a slow pace, during persistent infection [30,31]. Therefore, it must stay constantly ahead of the various arms of the immune response. For picornaviruses, the first to come into play are mediated by TLR3, TLR7, and the cytoplasmic helicase MDA-5. These receptors are located in endosomes/lysosomes, or in the cytoplasm in the case of MDA-5. They sense viral RNA and induce the nuclear translocation of transcription factors IRF-3, IRF-5, IRF-7, and NFkb and the expression of interferons and other cytokines such as TNF-alpha [32]. Clearly, the activation of this signaling cascade will depend on the organization of the cytoplasm of the infected cell. Nothing is known about the presence of the various components of these pathways in the cytoplasmic channels of myelin. However, one wonders about signalization cascades from the paranodal and internal loops to the very distant cell nucleus in cells with such unusual cytoplasmic organization. Is it possible that the interferon response to a virus that enters myelin from the axon is retarded compared to that in a more compact type of cell?

The main effector of the clearance of TMEV in genetically resistant mice is class I-restricted CD8+ cytotoxic T-lymphocytes recruited to the site of infection by the secretion of chemokines [33]. Could the myelin be a haven protected from cytotoxic T-lymphocytes? In the classical pathway the loading of class I molecules with peptides takes place in the endoplasmic reticulum. Since myelin cytoplasmic channels are far removed from the perinuclear endoplasmic reticulum of the oligodendrocyte cell body, viruses entering myelin from the axon may enjoy an environment where viral epitopes cannot be loaded on class I molecules efficiently. Furthermore, inefficient interferon secretion might delay the recruitment of cytotoxic T-lymphocytes. Also, by using axons to travel to distant sites within the CNS, the virus may escape from a local hostile environment, with cytokine secretion and inflammation, to new, virgin territories.

Finally, neutralizing antibodies participate in viral clearance in the CNS of resistant mouse strains [34]. Interestingly, the structure of the node of Ranvier, which has been extensively characterized at the molecular level [7], makes it impossible for immunoglobulins to diffuse through the septate-like junctions into the internodal space (between axon and myelin). Therefore, the virus might be protected from antibodies while it traffics from the axon to the myelin cytoplasm.

Clearly, the *shiverer* mutation, which causes a severe neurological deficit, was not selected for the advantage it gives mice by protecting them from TMEV persistent infection. However, it is possible that more innocuous polymorphisms of structural proteins have been selected because they interrupt the traffic of aggressive pathogens through a key organ such as the CNS.

Viruses have evolved an endless number of strategies to adapt to specific, highly specialized environments including the CNS. This paper points to the previously unrecognized use of axon/myelin interactions to foster viral persistence. It warrants looking for a similar role of myelin in the persistence in white matter of other viruses, including in humans.

## Materials and Methods

### Virus.

TMEV strain DA, grown on BHK-21 cells, had a titer of 2 ×10^8^ PFU/ml. Concentrated virus was used in some experiments. In this case, the clarified medium of infected BHK21 cells was layered on top of a 30% sucrose cushion and centrifuged at 17,000 rpm at 4 °C for 17 h in a Kontron TST 28.38 rotor. The virus pellet was re-suspended in 10 mM Tris HCl (pH 7.4). The titer of concentrated virus was 10^9^ PFU/ml.

### Mice.

C3HeB/FeJ and C3H *shi*/*shi* mice, referred to as “wild-type” and “*shiverer”* mice, respectively, in this article, were obtained from The Jackson Laboratory (http://www.jax.org). 4- to 6-wk-old, sex- and age-matched mice were used in all experiments. Anesthetized mice were inoculated intracranially with 10^6^ PFU of TMEV in 5 μl. Intra-vitreous injections were performed as follows: The conjunctiva was carefully cut with microsurgical scissors and forceps (Corneal) over approximately 1 mm, on the external side of the eye, to give access to the sclera. The sclera was punched with a 31-gauge needle. 2 ×10^6^ PFU of concentrated TMEV in 2 μl was injected between the crystalline lens and the retina with a microsyringe and a 33-gauge blunt-ended needle (Hamilton, http://www.hamiltoncompany.com). The virus had been mixed with a 1:100 dilution of a neutralizing anti-interferon type I serum (a gift from Ion Gresser, Curie Institute) in order to enhance viral replication in the retina.

### Total RNA extraction and reverse transcription.

Anesthetized mice were perfused with PBS through the left ventricle. Brains and spinal cords were dissected out and homogenized in Tri Reagent (Molecular Research Center, http://www.mrcgene.com) by passing the tissue through needles of decreasing diameter (18-, 21-, and 23-gauge). RNA isolation was performed according to the manufacturer's protocol. RNA was precipitated with ethanol, re-suspended in water, and its concentration was determined by spectrophotometry. 10 μg of extracted RNA was mixed with 3.5 μg of random hexamer primers p(dN)_6_ (Boehringer, http://www.boehringer-ingelheim.com), denatured for 20 min at 65 °C, then re-natured at room temperature to allow hybridization of the hexamer primers to the template. Reverse transcription was performed with AMV reverse transcriptase (Promega, http://www.promega.com) at 42 °C for 90 min.

To follow viral replication in retina, the eyes were dissected out and homogenized with a motor pellet pestle (Kimble/Kontes, http://www.kimble-kontes.com) in the Qiagen RNeasy RLT buffer (Qiagen, http://www1.qiagen.com). RNA extraction was performed according to Qiagen's protocol. The RNA extracted from one eye was mixed with 8 pmol of TM346 primer, in order to reverse transcribe viral minus-strand viral RNA only, and 8 pmol HPRTb primer (Table S3). The mixture was heated at 65 °C to denature RNA and DNA, and cooled at room temperature to allow hybridization of the primers to the RNA. Reverse transcription was performed with AMV reverse transcriptase at 42 °C for 90 min.

### Quantitative PCR.

Quantitative PCR was performed on the reverse transcription products using sequence specific Taqman probes, 2 × qPCR mastermix (Eurogentec, http://www.eurogentec.com) and an ABI Prism 7000 apparatus (Applied Biosystems, http://www.appliedbiosystems.com). Primers TM346 and TM347 were used to amplify reverse transcribed plus-strand and minus-strand viral RNA [35]. Primers HPRTa and HPRTb were used to amplify the reference housekeeping HPRT cDNA. Finally, the Taqman probes used were TM348 and HPRT-YY. All primers and probes are described in Table S3.

### Histology, immunofluorescence, and microscopy.

*Brain and spinal cord.* Mice were perfused with PBS followed by 4% para-formaldehyde (PFA). The brain and spinal cord were dissected out, post-fixed in 4% PFA for 1 h, incubated overnight in 15% sucrose before snap freezing in OCT. 10-μm cryostat sections were cut.

*Eye and optic nerve.* Mice were perfused with PBS followed by 4% PFA and were left intact for 1 h before dissecting out the eyes and optic nerves. Three holes were punched in the cornea with a 27-gauge needle to prevent drying up of the retina. Eyes and optic nerves were post-fixed for 2 h in 4% PFA, incubated for 2 h in 5% sucrose, and overnight in 15% sucrose before snap freezing in OCT. 5-μm cryostat sections were cut.

*Immunofluorescence.* Sections on slides were treated for 5 min with ice-cold ethanol or for 10 min with acetone at −20 °C. Sections were incubated for 2 h with the primary antibody diluted in PBS, 2% bovine serum albumin (BSA), 10% normal goat serum (NGS), 0.3% Triton X100, followed by washing and incubation for 1 h with the secondary antibody diluted in the same buffer. Slides were mounted in Vectashield with or without DAPI (Vector Laboratories, http://www.vectorlabs.com). Cells grown on cover slips were incubated for 1 h with the primary antibody diluted in PBS, 2% BSA, 10% NGS, 0.1% Triton X-100. Incubation with the secondary antibody was for 1 h in the same buffer but without Triton X100. Cover slips were mounted in fluoromount (EMS). All primary and secondary antibodies used in this work are described in Table S4. Immunofluorescence was examined with a Zeiss Axioplan 2 microscope (http://www.zeiss.com) equipped with the ApoTome device in order to obtain 0.7 μm thick optical sections. To examine marker colocalization, Z-stacks were built using the Axiovision 4.5 software. Marker specificity was confirmed by examining sections doubly stained for CNPase and GFAP (Video S1), CNPase and CD11b (Video S2), CNPase and PLP (Video S3), and CNPase and Olig-2 (Video S4).

### Bone marrow grafts.

Recipient mice were starved and treated with penicillin-streptomycin for 24 h before irradiation at 1,100 rad with a dose rate of 110 rad/min. Cells were flushed out of the femurs of donor mice and dissociated mechanically by passing through a 25-gauge needle. After counting, they were re-suspended at a concentration of 1.25 × 10^7^ cells/ml in PBS supplemented with 2% fetal calf serum (FCS). 200 μl of this cell suspension was injected intravenously into the retro-orbital sinus of irradiated recipient mice. The mice were kept for 7 wk in sterile conditions to allow reconstitution of their immune system. The level of chimerism was tested by PCR analysis on blood DNA. 80 μl of blood was taken from the retro-orbital sinus of each recipient mouse using a heparinized capillary. DNA was extracted from the blood as described [36]. DNA was amplified by 25 cycles of PCR, with an annealing temperature of 60 °C, using the WTf, WTr and SHIf, SHIr pairs of primers (Table S3). The amplicon obtained with wild-type DNA was a 216-bp long fragment located within the *shiverer* deletion. For *shiverer* DNA, a 315-bp fragment was amplified with primers located on either side of the deletion (Figure 1). The level of chimerism was estimated by comparing the PCR products with amplicons obtained from a series of reciprocal mixtures of wild-type and *shiverer* DNA (Figure 1).

### Oligodendrocyte primary cultures.

Oligodendrocyte primary cultures were generated with a protocol adapted from that of Lubetzki et al. [37]. Briefly, newborn mice were decapitated and brains were dissected out, chopped with a scalpel in HBSS buffered with 10 mM HEPES, digested with Trypsin-EDTA, then passed through a 70-μm nylon mesh. The cell suspension was layered on top of a 45% Percoll solution (Amersham, http://www.gehealthcare.com) and centrifuged at 23,500*g* for 45 min. The myelin layer was removed and the cellular layer was collected. Cells were washed, counted, and plated on poly-L lysine-coated glass cover slips at a density of 5 × 10^4^ cells per cover slip, in a 20-μl drop of DMEM (GIBCO, http://www.invitrogen.com) supplemented with 10% FCS (PAA). After incubation for 1 h at 37 °C, Bottenstein Sado medium supplemented with 10 ng/ml platelet-derived growth factor (PDGF)-AA (Euromedex, http://www.euromedex.com) was added to the cultures. Bottenstein Sado medium was DMEM-supplemented with 100 μg/ml apotransferrin, 900 ng/ml insulin, 100 μg/ml BSA, 60 ng/ml progesterone, 15 μg/ml putrescine, 40 ng/ml sodium selenite, 300 ng/ml of T3, and 400 ng/ml of T4 thyroid hormone (all from Sigma, http://www.sigmaaldrich.com), 0.5% FCS. The medium was changed once after 4 d. After 1 wk, it was replaced by Bottenstein-Sado medium without PDGF to trigger the differentiation of oligodendrocyte progenitors. The Bottenstein-Sado medium without PDGF was changed twice a week for 2 wk. The cultures were then infected with 500 PFU/cell of TMEV. At different times p.i., the cover slips were washed three times in PBS, fixed for 15 min with 4% PFA, and washed again three times before performing immunofluorescence.

### Analysis of CNS inflammatory cells.

Anesthetized mice were perfused with PBS. The brains were dissected out, chopped with a scalpel in HBSS buffered with HEPES 10 mM, digested for 30 min at 37 °C with a mixture of 100 μg/ml DnaseI (Boehringer), 1 mg/ml of Collagenase D (Sigma), and 100 ng/ml of TLCK (Sigma). The cell suspension was passed through a 70-μm mesh. Cells were pelleted, re-suspended in 30% Percoll, and the cell suspension was layered on top of a 70% Percoll cushion. After centrifugation at 900*g* at room temperature for 20 min, the layer of inflammatory cells was collected and washed once in PBS. Inflammatory cells were incubated for 10 min at room temperature with an anti-CD16/CD32 antibody (Pharmingen, http://www.bdbiosciences.com) diluted 1:25 in PBS in order to block Fc receptors. The cells were then incubated for 30 min at 4 °C with 1:100 dilutions of FITC coupled anti-CD11c antibody, FITC coupled anti-CD4 antibody, PE coupled anti-CD3 antibody, PE coupled anti-CD45 antibody, PerCP coupled anti-CD45 antibody, PerCP coupled anti-CD8 antibody, Alexa 647 coupled anti-CD11b antibody, or Alexa 647 coupled anti-CD19 antibody (all from Pharmingen). Cells were washed and re-suspended in PFA 2% for flow cytometry analysis. For double staining for viral capsid antigens, the cells were fixed and permeabilized for 4 min at 4 °C in Cytofix/Cytoperm (Becton Dickinson, http://www.bdbiosciences.com), washed in PBS 2% FCS, and incubated for 1 h at 4 °C with a 1/20 dilution in PBS, 2% FCS of a hyper-immune serum directed against TMEV capsid antigens. After one wash in PBS, 2% FCS, cells were incubated for 1 h at 4 °C with an anti-rabbit IgG antibody coupled to Alexa 488 (Molecular Probes, http://probes.invitrogen.com) diluted 1:200. Cells were washed in PBS, 2% FCS, and re-suspended in 2% PFA before flow cytometry analysis. Flow cytometry analysis was done with a FACSCalibur (Becton Dickinson) with the Cellquest software.

### Infectious center assays.

CNS inflammatory cells prepared as described above were washed twice in PBS, counted, and a 1:100 dilution of a neutralizing serum directed against the viral capsid was added to the cell suspension, in order to neutralize free viral particles and to monitor only the virus producing cells. 4 × 10^4^, 4 × 10^3^, and 4 × 10^2^ cells were plated on BHK-21 monolayers in 6-well plates and incubated for 1 h 30 at 37 °C. The inoculum was removed (the inflammatory cells adhered to the reporter cell layer) and DMEM containing 1% FCS and 0.8% agarose was added on top of the cell layer. After 3 d, the cells were fixed with 5% formaldehyde for 1 h at room temperature, the agarose was removed, the cells were stained with crystal violet, and the plaques were counted.

## Supporting Information

Figure S1 Inflammatory Cells in the CNS of Mice Injected with poly(I:C) or Infected with TMEV (A) Entry of inflammatory cells in the CNS following poly(I:C) injection. Mice were injected intracranially with 10 μg of poly(I:C). After 24 h, inflammatory cells were extracted and stained for CD11b and CD45. The ordinate shows the ratio (number of CD11b+CD45^hi^ cell)/(number of CD11b+CD45^lo^ cells). Each circle corresponds to an individual mouse.
(B) Mice were inoculated intracranially with 4 × 10^6^ PFU of TMEV, sacrificed 5 d later, and inflammatory cells were extracted from brain. The percentage of CD11b+CD45^hi^, CD11b+CD45^lo^, CD3+CD4+, CD3+CD8+, CD19+, and CD11c+ cells was measured by flow cytometry. For each category of cell, the ordinate shows the ratio of their percentage in the population over the percentage of CD11b+CD45^lo^ cells (microglial cells), which is a constant. No statistically significant difference (Mann-Whitney test, *p* > 0.1) between cells from wild-type and *shiverer* mice was found for all cell types analyzed.
(41 KB PPT)

Figure S2 Viral Loads in the CNS of Wild-Type and *shiverer* Mice at Different Times p.i. Mice were inoculated intracranially with 10^6^ PFU of TMEV. Total RNA was extracted from brain and spinal cord at different times p.i. The amount of viral RNA and of HPRT mRNA, a reference housekeeping mRNA, were measured by quantitative RT-PCR. The ordinate shows the ratio (amount of viral RNA)/(amount of HPRT mRNA). Abscissa, day post-inoculation. (A) Viral RNA in brain. (B) Viral RNA in spinal cord. Each circle corresponds to an individual mouse.
(39 KB PPT)

Figure S3 Diagrammatic View of CNS Myelin CNS myelin is an extension of the cytoplasmic membrane of the oligodendrocyte that wraps itself many times around axons. Most of the cytoplasm is extruded from myelin (in gray); however, cytoplasmic channels that connect the myelin sheath to the oligodendrocyte cell body remain. These channels contact the axon at the level of nodes of Ranvier, forming the so-called paranodal loops, as well as along the internode.
(252 KB PPT)

Table S1 Raw Data for the Quantitative RT-PCR Experiments Presented in Figure 1
(16 KB XLS)

Table S2 Raw Data for the Quantitative RT-PCR Experiments Presented in Figure S2
(9 KB XLS)

Table S3 Primers and Probes Used for RT and PCR (16 KB XLS)

Table S4 Antibodies Used for Immunofluorescence Staining (16 KB PPT)

Video S1 Frozen Section of Optic Nerve of a Mouse Inoculated in the Vitreous Chamber with TMEV and Sacrificed 4 d Later The section was doubly stained for CNPase (green) and GFAP (red). Nuclei were stained blue with DAPI. The section was examined with a Zeiss Axioplan 2 microscope equipped with the Apotome device in order to obtain 0.7 μm thick optical sections. The 3-D reconstruction was built using the Axiovision 4.5 software. The markers are not expressed by the same cells.
(8.4 MB MOV)

Video S2 Frozen Section of Optic Nerve of a Mouse Inoculated in the Vitreous Chamber with TMEV and Sacrificed 4 d Later The section was doubly stained for CNPase (green) and CD11b (red). Nuclei were stained blue with DAPI. The section was analyzed as described in the legend of Video S1. The two markers are not expressed by the same cells.
(8.2 MB MOV)

Video S3 Frozen Section of Optic Nerve of a Mouse Inoculated in the Vitreous Chamber with TMEV and Sacrificed 4 d Later The section was doubly stained for CNPase (green) and PLP (red). Nuclei were stained blue with DAPI. The Z-stack was obtained using the Axiovision 4.5 software. The video illustrates the simultaneous expression of both markers in oligodendrocytes, often close to the cytoplasmic membrane, as well as in some cytoplasmic extensions of oligodendrocytes and in myelin.
(8.9 MB MOV)

Video S4 Frozen Section of Optic Nerve of a Mouse Inoculated in the Vitreous Chamber with TMEV and Sacrificed 4 d Later The section was doubly stained for CNPase (green) and Olig-2 (red). Nuclei were stained blue with DAPI. The Z-stack was obtained using the Axiovision 4.5 software. The video illustrates CNPase-positive cells whose nucleus contains the oligodendrocyte-specific Olig-2 marker.
(3.7 MB MOV)

Video S5 Frozen Section of Optic Nerve of a Mouse Inoculated in the Vitreous Chamber with TMEV and Sacrificed 4 d Later The section was doubly stained for CNPase (green) and TMEV capsid antigen (red). Nuclei were stained blue with DAPI. The section was examined with a Zeiss Axioplan 2 microscope equipped with the Apotome device in order to obtain 0.7 μm thick optical sections. The video illustrates how optical section Z-stacks were used to assess colocalization of fluorescent markers. The video shows an example of infected CNPase-positive cell (oligodendrocyte).
(8.3 MB MOV)

Video S6 Frozen Section of Optic Nerve of a Mouse Inoculated in the Vitreous Chamber with TMEV and Sacrificed 4 d Later The section was doubly stained for GFAP (green) and TMEV capsid antigen (red). Nuclei were stained blue with DAPI. The section was examined with a Zeiss Axioplan 2 microscope equipped with the Apotome device in order to obtain 0.7 μm thick optical sections. The video illustrates how optical section Z-stacks were used to assess colocalization of fluorescent markers. The video shows an example of infected GFAP-positive cell (astrocyte).
(5.0 MB MOV)

## Abbreviations

CNPase: 2′,3′-cyclic nucleotide 3′ phosphohydrolase
CNS: central nervous system
DAPI: di-aminido phenylindol
GFAP: glial fibrillary acidic protein
LGN: lateral geniculate nucleus
MBP: myelin basic protein
MS: multiple sclerosis
p.i.: postinoculation
PBL: peripheral blood lymphocyte
PDGF: platelet derived growth factor
PFU: plaque-forming unit
PLP: proteolipid protein
poly(I:C): polyinosinic:polycytidilic acid
shi: *shiverer;*
TLR: toll-like receptor
TMEV: Theiler's murine encephalomyelitis virus
